# Comparison of the efficacy between concurrent chemoradiotherapy with or without adjuvant chemotherapy and intensity-modulated radiotherapy alone for stage II nasopharyngeal carcinoma

**DOI:** 10.18632/oncotarget.11978

**Published:** 2016-09-12

**Authors:** Kai-Hua Chen, Xiao-Dong Zhu, Ling Li, Song Qu, Zhen-Qiang Liang, Xia Liang, Xin-Bin Pan, Zhong-Guo Liang, Yan-Ming Jiang

**Affiliations:** ^1^ Department of Radiation Oncology, Affiliated Tumor Hospital of Guangxi Medical University, Cancer Institute of Guangxi Zhuang Autonomous Region, Nanning, Guangxi, China; ^2^ Key Laboratory of Early Prevention and Treatment for Regional High Frequency Tumor (Guangxi Medical University), Ministry of Education, Nanning, Guangxi, China; ^3^ Guangxi Key Laboratory of Early Prevention and Treatment for Regional High Frequency Tumor (Guangxi Medical University, Nanning, Guangxi, China

**Keywords:** nasopharyngeal neoplasm, stage II, concurrent chemoradiotherapy, adjuvant chemotherapy, intensity-modulated radiotherapy, prognosis

## Abstract

**Objective:**

This study aimed to explore whether concurrent chemoradiotherapy (CCRT) with or without Adjuvant Chemotherapy (AC) could improved the survival in stage II nasopharyngeal carcinoma (NPC).

**Methods:**

Patients with stage II NPC treated with CCRT (*n*=80) or CCRT+AC (*n*=40) or IMRT alone (*n*=42) between January 2007 and September 2014 were retrospectively analyzed. The three patient groups were matched based on prognostic factors. All patients were treated with IMRT. The endpoints were overall survival (OS), distant metastasis-free survival (DMFS), locoregional relapse-free survival (LRRFS), and failure-free survival (FFS). The treatment-related acute toxicity reactions between the three groups were compared also.

**Results:**

The three groups indicated similar outcomes: survival of the CCRT group, CCRT+AC group and RT alone group were (93.9%, 95.0%, 95.2%, *P*=0.937) for OS, (96.8%, 94.9%, 93.0%, *P*=0.756) for LRRFS, (91.1%, 97.5%, 100%, *P*=0.185) for DMFS and (84.9%, 92.5%, 93.0%, *P*=0.597) for FFS. Both the univariate and multivariate analysis indicated that older age predicted lower LRRFS and FFS. The CCRT and CCRT+AC groups showed more acute toxicity reactions, especially in bone marrow suppression, Liver dysfunction, gastrointestinal reactions (nausea/vomiting) and weight loss.

**Conclusion:**

CCRT with/without AC could not improve the survival conditions of patients with stage II NPC, but remarkably increased treatment-associated acute toxic reactions when compared with IMRT alone.

## INTRODUCTION

Nasopharyngeal carcinoma (NPC) is the malignant tumor with high incidence in southern part of China as well as Southeast Asia [1]. The major treatment of which is radiotherapy at present. There have been appreciable quantities of prospective studies [2-6] and Meta-analysis [7] verifying that concurrent chemoradiotherapy (CCRT) with/without adjuvant chemotherapy (AC) have better therapeutic effects on improving locally advanced NPC than radiotherapy alone. However, no definitive conclusion has been reached yet regarding whether chemotherapy is required in treating stage II NPC. The guideline of American National Comprehensive Cancer Network (NCCN) suggests CCRT with/without AC for patients with stage II NPC; however, the guideline lacks potent evidence-based medicine evidence. Chinese Anti-cancer Association (CACA) recommends radiotherapy alone for patients with T2N0M0, but there are no clearly established criteria for T1-2N1M0 cases, which can either be treated with radiotherapy alone or with comprehensive treatments of radiotherapy and chemotherapy.

There have been researches demonstrating that CCRT can improve the survival for patients with stage II NPC in the time of conventional radiotherapy (CRT) [8-10], however, only one of them is prospective study so far [10]; while in the era of intensity-modulated radiotherapy (IMRT), many studies discover that IMRT alone has achieved superior therapeutic effects on treating stage II NPC [11-13]. Luo et al [14] revealed in their research that CCRT had higher 3-year overall survival than IMRT alone in patients with early-stage NPC (100.0% vs 81.4%, *P*=0.04), however, the cases whose pathological types were dominated by WHO II type (71%) mainly came from the non-high prevalence areas of NPC. In addition, as was reported in a multi-center study from Korea [15], CCRT improved the 5-year locoregional relapse-free survival (LRRFS) as well as the progression free survival (PFS) for patients with stage II NPC, however, 43.5% (60/138) of the cases in the study adopted CRT, and the WHO I and II types accounted for 42% (58/138) of the pathological types. In 2015, there were several studies demonstrating that CCRT could not improve the prognosis for patients with early-stage NPC but increased the treatment-associated acute toxic reactions when compared with IMRT alone [16-18].

The article retrospectively analyzed the survival conditions of three groups of patients with stage II NPC that received CCRT, CCRT+AC, and IMRT alone, respectively, during the same period in our hospital, and probed into the effects of chemotherapy on patients with stage II NPC who received IMRT.

## MATERIALS AND METHODS

### Patients and patient workup

The clinical data of the untreated NPC patients that received IMRT in the affiliated Tumor Hospital of Guangxi Medical University from January, 2007 to September, 2014 were collected to conduct a restaging in accordance with the 7^th^ edition American Joint Committee on Cancer/Union for International Cancer Control (AJCC/UICC) staging system [19, 20], and all together 162 cases of patients with stage II NPC were included. All patients were clearly diagnosed pathologically, and received examination such as nasopharyngoscope, nasopharyngeal and neck magnetic resonance imaging (MRI), chest computed tomography (CT), as well as whole body bone scanning before treatment.

### Treatment protocols

#### Radiotherapy

All patients were treated with IMRT. 80 cases received CCRT, 40 received CCRT with AC, and 42 received IMRT alone. The gross tumor volume of nasopharynx (GTVnx) included the primary gross tumor volume and involved retropharyngeal nodes. The positive cervical lymph nodes were delineated as gross tumor volume of involved cervical lymph nodes (GTVnd). Both the GTVnx and GTVnd were determined based on MRI, clinical and nasopharyngoscope findings. High-risk clinical target volume (CTV-1) was defined as the area from 5-10 mm outside the GTV. The low-risk clinical target volume (CTV2) was defined as the margin from 5-10 mm around CTV1 and the lymphatic drainage area (Levels II, III, IV and V). The planning target volumes (PTV) were defined by enlarging 3 mm margin to the GTV or CTV. The prescribed doses were as follows: 68-70 Gy/30-31f for PGTVnx and PGTVnd, 60-66Gy/30-31f for PCTV1, and 50-56 Gy/30-31f for PCTV2, with one irradiation each day for five times a week.

#### Chemotherapy

The concurrent chemotherapy regimen was 80-100 mg/m^2^ of cisplatin altogether for 3 days in a cycle of 21 days for 2 to 3 cycles. The adjuvant chemotherapy regimen was 80 mg/m^2^ of cisplatin for 1 day, 600 mg/m^2^/d of 5-fluorouracil for continuous intravenous infusion 120h in a cycle of 28 days for 2 to 3 cycles.

### Endpoints and toxicity assessments

The survival time was calculated from the date of treatment; and the overall survival (OS), locoregional relapse-free survival (LRRFS), distant metastasis-free survival (DMFS), as well as failure-free survival (FFS) were treated as the endpoints, among which OS, LRRFS and DMFS referred to the duration from the date of treatment to the date of death, nasopharyngeal or locoregional lymph node relapse, and distant metastasis, respectively; while FFS was the duration from the date of treatment to the date of relapse, metastasis or death due to any cause. The Common Terminology Criteria for Adverse Events (CTCAE, v4.0) [21] was applied in observing treatment-associated toxicity reactions, and the indicators for further observation included: bone marrow suppression, liver and renal dysfunction, gastrointestinal reactions and weight loss.

### Follow-up

The patients were followed up by means of out-patient visits, letters and phone calls after treatment ended. The patients were re-checked once every three months within the 1^st^ to 2^nd^ year, once every 6 months within the 3^rd^ to 5^th^ year, and once every year afterwards; and the re-check items included physical examination, nasopharyngoscope, chest radiography or CT, abdominal B ultrasound, nasopharyngeal and neck MRI, as well as whole body bone scanning when necessary.

### Statistical analysis

The SPSS18.0 software was applied in data analysis and process, chi-square test or Fisher's exact test was utilized to compare the general clinical data in pairs; Kaplan-Meier method was adopted to calculate survival rates; log-rank test was employed for pairwise comparison among groups; and COX regression analysis was used for univariate and multivariate analysis. Non-parametric Kruskal-Wallis test was introduced in comparing the acute toxic reactions, and all differences with P<0.05 were deemed as statistically significant.

## RESULTS

### Patient characteristics

Of the 162 cases of patients with stage II NPC, 80 cases received CCRT, 40 received CCRT with AC, and 42 received IMRT alone. There were no statistically significant differences of the proportional distribution of age, sex, as well as pathological types and T stages (all *P*>0.05), while the differences of the proportional distribution of N stages and clinical stages in the three groups were statistically significant (all *P*<0.001). The comparison of the balance of patient characteristics in the three groups was shown in Table 1.

**Table 1:** Characteristics of the three groups of patients

Variate	CCRT	CCRT+AC	IMRT alone	*P* value
Age				0.639
≤45	47	25	22	
>45	33	15	20	
Sex				0.400
female	23	10	16	
male	57	30	26	
Pathology (WHO)				0.189
II type	7	8	7	
III type	73	32	35	
T stage				0.548
T1	14	4	6	
T2	66	36	36	
N stage				P<0.001
N0	6	1	18	
N1	74	39	24	
Clinical stage				P<0.001
T2N0M0	6	1	18	
T1N1M0	14	4	6	
T2N1M0	60	35	18	

### Follow-up results

The endpoint of the follow-up was December 10^th^, 2015; the median follow-up period was 56 months (9 to 100 months), and 6 cases of patients lost to follow-up, with the follow-up rate of 96.3%. There were altogether 5 cases of death among all patients, all of which were tumor-associated death, and there was no statistically significant difference of event distribution in patients of the three groups, the detailed results of which were shown in Table 2.

**Table 2:** Follow-up results of the three groups of patients

Endpoint	Group	No.	Event (n)	*P* value
Death	CCRT	80	2	0.937
	CCRT+AC	40	2	
	IMRT alone	42	1	
Locoregional relapse	CCRT	80	2	0.756
	CCRT+AC	40	2	
	IMRT alone	42	3	
Distant metastasis	CCRT	80	4	0.185
	CCRT+AC	40	1	
	IMRT alone	42	0	

### Survival outcomes

**Figure 1:**
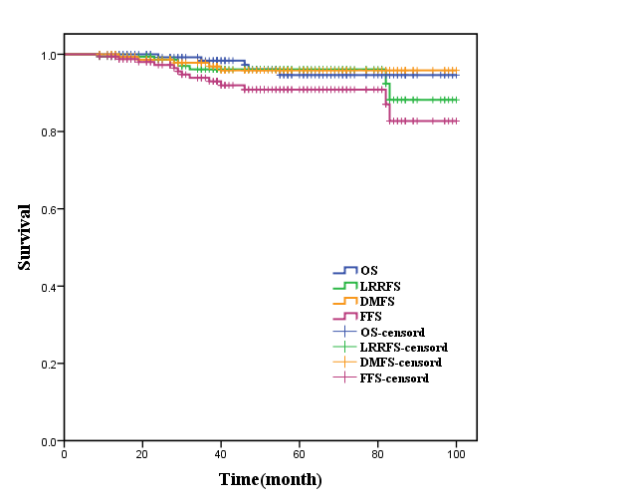
Survival curves for entire patients

The 5-year OS, LRRFS, DMFS and FFS of all patients were 94.6%, 96.1%, 95.8% and 89.5%, respectively, and the survival curves were shown in Figure 1; while the 5-year OS, LRRFS, DMFS and FFS of the three subgroups T2N0M0, T1N1M0 and T2N1M0 were (92.9%, 100.0%, 94.3%, *P*=0.679), (95.7%, 100.0%, 95.5%, *P*=0.563), (95.5%, 100.0%, 95.1%, *P*=0.683), and (80.5%, 100.0%, 89.1%, *P*=0.365), respectively.

**Figure 2:**
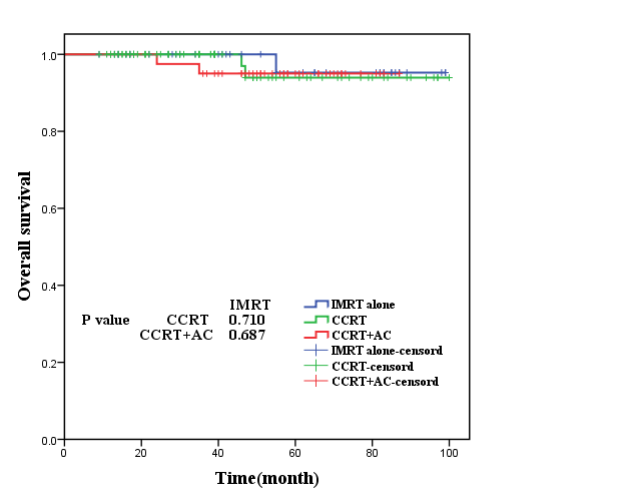
Overall survival for the three groups of patients

**Figure 3:**
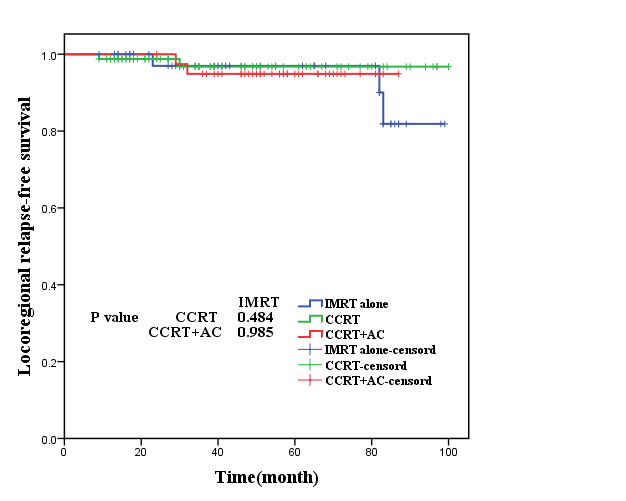
Locoregional relapse-free survival for the three groups of patients

**Figure 4:**
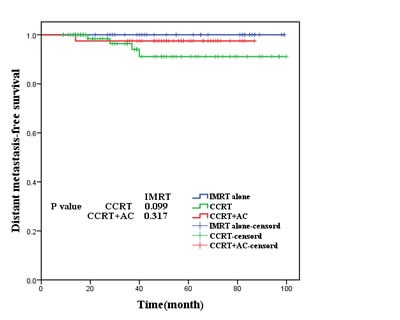
Distant metastasis-free survival for the three groups of patients

**Figure 5:**
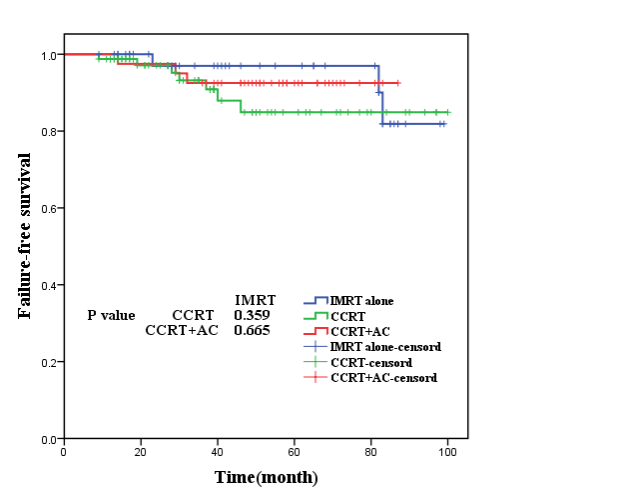
Failure-free survival for the three groups of patients

As was shown in Figures 2,3, 4, 5, the 5-year OS, LRRFS, DMFS and FFS in the CCRT group, CCRT+AC group, as well as IMRT alone group were (93.9%, 95.0%, 95.2%, P=0.937), (96.8%, 94.9%, 93.0% (*P*=0.756), (91.1%, 97.5%, 100.0%, *P*=0.185), and (84.9%, 92.5%, and 93.0%, *P*=0.597), respectively, with no statistically significant difference in pairwise comparison among groups.

### Univariate analysis

Both the results of univariate and multivariate analysis demonstrated that the effects of age on patients' LRRFS and FFS were statistically significant, which meant that LRRFS and FFS tended to decrease with the increase in age. The results of univariate and multivariate analysis were shown in Table 3.

**Table 3:** Univariate and multivariate analysis of prognostic factors

Variate	*P* value								
	Univariate analysis					Multivariate analysis			
	OS	LRRFS	DMFS	FFS		OS	LRRFS	DMFS	FFS
Age	0.792	0.020	0.453	0.042		0.796	0.002	0.568	0.014
Sex	0.370	0.836	0.385	0.205		0.126	0.836	0.142	0.188
Pathological type	0.565	0.537	0.520	0.368		0.367	0.335	0.356	0.158
T stage	0.577	0.520	0.577	0.367		0.384	0.316	0.383	0.158
N stage	0.965	0.577	0.899	0.674		0.965	0.574	0.899	0.673
Clinical stage	0.443	0.538	0.466	0.281		0.744	0.885	0.799	0.847
Treatment	0.732	0.814	0.668	0.933		0.731	0.814	0.667	0.933

### Acute toxicity reactions

No grade 4 or fatal toxicity reactions were seen in all patients, while grade 3 toxicity reactions could be seen in a few cases. The article revealed that the differences of the degrees of toxicity reactions such as leukopenia, neutropenia, anemia, liver dysfunction, gastrointestinal reactions (nausea/vomiting), and weight loss were statistically significant among the three groups patients, while there were no statistically significant differences of the degrees of thrombocytopenia and renal dysfunction among the three groups patients, with the detailed pairwise comparison being shown in Table 4.

**Table 4:** Acute toxicity reactions of the three groups of patients

Toxicity reaction	CCRT	CCRT+AC	IMRT alone	*P* value
leukopenia	a	a		<0.001
0	18	4	30	
1	20	9	8	
2	29	18	3	
3	13	9	1	
neutropenia	a	a		<0.001
0	37	10	39	
1	21	11	2	
2	17	6	0	
3	5	3	1	
anemia	a	a		0.002
0	46	23	37	
1	18	14	3	
2	14	3	2	
3	2	0	0	
thrombocytopenia				0.093
0	68	36	41	
1	7	2	1	
2	2	2	0	
3	3	0	0	
liver dysfunction	a	a		<0.001
0	64	20	39	
1	13	16	2	
2	3	2	1	
3	0	1	0	
renal dysfunction				0.108
0	79	38	41	
1	10	2	1	
gastrointestinal	a	a		<0.001
0	8	7	33	
1	18	5	5	
2	47	27	4	
3	7	1	0	
weight loss	a	a b		
0	22	4	25	<0.001
1	35	17	10	
2	23	18	7	
3	0	1	0	

a indicated that the difference of the acute toxicity reaction degree between that group and the IMRT alone group was statistically significant (p<0.05), while b meant that the difference of the acute toxicity reaction degree between that group and the CCRT group was statistically significant (p<0.05).

## DISCUSSION

The treatment mode of stage II NPC is quite controversial at present. The results of this article suggested that, CCRT with/without AC could not improve the survival of patients with stage II NPC, but remarkably increased treatment-associated acute toxicity reactions when compared with IMRT alone.

A large number of studies indicated that the main mode of treatment failure in NPC at the present stage was distant metastasis [22-25], and improving the local control rate was one of the objectives of comprehensive treatments of chemoradiotherapy. Since the 0099 experiment in America reports that the regimen of CCRT combined with AC can improve the OS and the PFS for patients with locally advanced NPC [26], the regimen has been gradually considered to be the standard treatment protocol for locally advanced NPC. At present, the NCCN guide recommends CCRT with/without AC for patients with stage II to IVb NPC. Xiao et al [23] showed in their research that T2, N1 were the high risk factors of distant metastasis for patients with early stage NPC, and the co-existence of the two factors resulted in the distinctly decreased DMFS in T2N1M0 patients than in T1-2N0M0 and T1N1M0 patients (*P*<0.05). Several previous studies reported the positive role of CCRT in treating patients with stage II NPC [8-10]. Though this seemed to illustrate that it was reasonable to apply CCRT in stage II nasopharyngeal carcinoma or its subgroups, it was undeniable that the treatment approaches that all cases in the above-mentioned studies received were conventional radiotherapy. Is there still a need to combine chemotherapy in treating stage II NPC in the era of IMRT?

Su et al [13] studied the survival conditions of 198 cases of patients with early stage NPC that received IMRT alone, the results of which revealed that the 5-year DMFS of T2N0M0, T1N1M0 and T2N1M0 were 98.8%, 100% and 93.8%, respectively (*P*>0.05), indicating few cases of distant metastasis in the three subgroups after treated with IMRT alone and no obvious differences among groups. In our research, the subgroup analysis of all cases presented similar results, with the 5-year DMFS of T2N0M0, T1N1M0 and T2N1M0 being 95.5%, 100.0% and 95.1%, respectively (*P*=0.683). It was a pity that no subgroup analysis that aimed at the IMRT alone group was conducted due to the small sample size of IMRT alone cases in the research.

The results in our research showed that the 5-year LRRFS and DMFS of all patients were 96.1% and 95.8%, respectively; while the LRRFS and DMFS in the CCRT group and the IMRT alone group were (96.8% vs 93.0%,*P*=0.484), and (91.1% vs 100.0%,*P*=0.099), respectively, which was similar to the previous relevant reports. In 2015, Su et al [16] reported that the 5-year LRRFS and DMFS of patients with stage II NPC that received CCRT and IMRT alone were (94.8% vs 89.3%, *P*=0.167) and (93.4% vs 97.5%, *P*=0.349), respectively, with no statistically significant difference. Zhang et al [17] showed in their research that the survival conditions of stage II NPC treated with IMRT alone were similar to those treated with CCRT. Xu et al [18] compared the effects of CCRT and IMRT alone on the survival of T1-2N1M0, the results of which displayed that the LRRFS and DMFS of patients in the two groups were (92.3% vs 95.0%, *P*=0.885), and (97.6% vs 91.7%, *P*=0.631), respectively, and no superiority of concurrent chemotherapy was shown. Our center reported previously that CCRT could not improve the prognosis for patients with stage II NPC relative to radiotherapy alone [27]; however, the downside was that only 54.2% (58/107) cases in the research received IMRT. According to the NCCN guideline, we collected patients in the CCRT with AC group to compare with those in the IMRT alone group, the results of which also showed that CCRT with AC could not improve the survival of patients with stage II NPC. To sum up, IMRT alone had satisfying effects on patients with stage II NPC, with limited opportunity for increasing the survival rate, leading to the failure for chemotherapy to highlight its advantage in enhancing the local control rate.

In recent years, though Luo et al [14] and Kang et al [15] reported that CCRT could boost the survival rate of patients with stage II NPC, the two studies came from the non-high prevalence areas of NPC, with WHO I or II type accounting for an overwhelming part in the pathological types; besides, only 56.5% (78/138) cases in Kang's study received IMRT. As a result, the two studies had limited therapeutic reference value for stage II NPC in high prevalence areas under the technical condition of IMRT, while whether CCRT was required for treating stage II NPC in non-high prevalence areas remained to be further explored.

The results of our research displayed that a majority of the acute toxicity reactions of all patients were grade 1 and 2, a few cases developed grade 3 acute toxicity reactions, and no grade 4 or fetal acute toxicity reactions occurred. The degrees of acute toxic reactions were notably higher in the CCRT group and the CCRT+AC group than in the IMRT alone group, which mainly manifested in aspects like leucopenia, neutropenia, anemia, liver dysfunction, gastrointestinal reaction, and weight loss; while there were no statistically significant differences of the degrees of renal dysfunction and thrombocytopenia. When compared the CCRT group with the CCRT+AC group, the degrees of liver dysfunction and weight loss were more evident in the latter. A meta-analysis which contained 13 randomized controlled trials [28] revealed that cisplatin-based chemoradiotherapy increased the treatment-associated death (1.7% vs 0.8%) and severe toxicity reactions relative to radiotherapy alone. As was reported in previous research [16-18], acute toxic reactions were not associated with the degree of weight loss. In our research, the proportions of patients with grade 1 to 2 weight loss in the CCRT group, the CCRT+AC group, and the IMRT alone group were 72.5% (58/80), 87.5% (35/40), and 40.5% (17/42), respectively. Shen et al [29] indicated in their research that high degree of weight loss (≥5%) was the independent prognostic factors of decreased OS for NPC patients with normal or low body weight before treatment. It appear that chemotherapy can not manifest its positive effects on the survival of patients with stage II NPC that receive IMRT, but apparently increase the treatment-associated acute toxic reactions, consequently, clinicians have to take the advantages and disadvantages of chemotherapy into careful consideration, and prepare treatment protocols that are more beneficial to patients with stage II NPC.

The differences of distribution of N stages and clinical stages among the three groups of patients in our research were of statistical significance; however, univariate and multivariate analysis indicated that N stages and clinical stages were not the influence factors of prognosis, it was of certain clinical value when taking the relative balanced distribution of other factors among the three groups into consideration. Certainly, there were still drawbacks in this research: firstly, it was a single-center retrospective study, which together with the small sample size and limited follow-up period, leading to little outcome; and secondly, late toxicity reactions could not be completely collected for analysis as a result of follow-up limit.

In conclusion, CCRT with/without AC could not improve the survival conditions of patients with stage II NPC, but remarkably increased treatment-associated acute toxic reactions when compared with IMRT alone. It is without any doubt that multi-center prospective randomized controlled trials should be carried out for further verification.
